# A rapid colorimetric lateral flow test strip for detection of live *Salmonella* Enteritidis using whole phage as a specific binder

**DOI:** 10.3389/fmicb.2022.1008817

**Published:** 2022-09-29

**Authors:** Ratthaphol Charlermroj, Manlika Makornwattana, Sudtida Phuengwas, Nitsara Karoonuthaisiri

**Affiliations:** ^1^National Center for Genetic Engineering and Biotechnology (BIOTEC), National Science and Technology Development Agency (NSTDA), Pathum Thani, Thailand; ^2^International Joint Research Center on Food Security, Pathum Thani, Thailand; ^3^Institute for Global Food Security, Queen’s University Belfast, Belfast, United Kingdom

**Keywords:** phage-derived antibody fragment, foodborne pathogen, *Salmonella* Enteritidis, lateral flow assay, colorimetric assay

## Abstract

Specific antibodies are essential components of immunoassay, which can be applied for the detection of pathogens. However, producing an antibody specific to live bacterial pathogens by the classical method of immunizing animals with live pathogens can be impractical. Phage display technology is an effective alternative method to obtain antibodies with the desired specificity against selected antigenic molecules. In this study, we demonstrated the power of a microarray-based technique for obtaining specific phage-derived antibody fragments against *Salmonella*, an important foodborne pathogen. The selected phage-displayed antibody fragments were subsequently employed to develop a lateral flow test strip assay for the detection of live *Salmonella*. The test strips showed specificity to *Salmonella* Enteritidis without cross-reactivity to eight serovars of *Salmonella* or other bacteria strains. The test strip assay requires 15 min, whereas the conventional biochemical and serological confirmation test requires at least 24 h. The microarray screening technique for specific phage-based binders and the test strip method can be further applied to other foodborne pathogens.

## Introduction

*Salmonella* bacterial species are causative agents of foodborne illness in humans and animals, which are commonly found in many types of food such as pork, eggs, poultry, seafood, unpasteurized dairy products, and vegetables (Jackson et al., 2013; Gu et al., 2018). The standard methods for detecting *Salmonella* are based on culturing techniques including pre-enrichment, selective-enrichment, and confirmation with biochemical tests, following procedures outlined by the International Organization for Standardization (ISO 6579) or Bacteriological Analytical Manual (BAM). These methods can detect low numbers or injured viable *Salmonella*; however, they are time-consuming and laborious. Thus, rapid and accurate methods are required for detecting foodborne pathogens (Law et al., 2015).

Methods have been developed to detect viable bacterial cells using fluorescent dyes such as SYTO 9 and propidium iodide (Ou et al., 2019), and mammalian cell-based immunoassay (Xu et al., 2020). Although they can detect or differentiate live cells from dead cells, these methods require many steps and special equipment. These shortcomings could be addressed by immuno-based lateral flow assays, which are more rapid, simple, and affordable. However, to our knowledge, there is no report of an immuno-based lateral flow method capable of discriminating viable from dead bacteria cells. The major challenge for applying lateral flow assays for detecting viable bacteria is the requirement for an antibody that binds specifically to viable bacterial cells of interest, but not to injured or non-viable cells.

The traditional method of producing an antibody for immunoassay relies on an *in vivo* immune response from an antigen. The success of antibody production depends on the antigen characteristics such as types of immunogens, antigenicity, and antigen dosing. Alternatively, antibodies can be produced by phage display technology, which can identify binders to antigens regardless of their immunogenic properties, thus allowing the selection of binders against self-antigens, toxic, unstable, and non-immunogenic antigens (Frenzel et al., 2016). This technology also facilitates genetic engineering of the binding sites to improve affinity and specificity. Its advantages over the traditional *in vivo* antibody production method have fostered applications ranging from epitope mapping (Spillner et al., 2003; Youn et al., 2004), the detection of bacteria and viruses (Ferrer and Harrison, 1999; Yang et al., 2003; Morton et al., 2013b; Karoonuthaisiri et al., 2014; Wang et al., 2014; Niyomdecha et al., 2018), protein domains (Christ and Winter, 2006), and small molecules (Zhao et al., 2005; Qi et al., 2008).

Given the power of phage technology, this study aimed to (1) develop a bacterial microarray method to speed up the process of screening and selecting phage clones expressing specific antibody fragments and (2) utilize the selected phage clones for developing a rapid lateral flow detection method for live *Salmonella* Enteritidis.

## Materials and methods

### Bacteria, antibodies, and phage clones

All bacteria in Table 1, except for *Campylobacter* spp., were inoculated from a single colony grown in a LB agar plate and cultured in 10 mL of 2xYT medium (16 g/L tryptone, 10 g/L yeast extract, and 5 g/L NaCl) at 37^°^C, 250 rpm for 16–18 h. *Campylobacter* spp. were cultured in 10 mL of Campylobacter Enrichment Broth (CEB) supplemented with 20 mg/L cefoperazone, 20 mg/L vancomycin, 20 mg/L trimethoprim, and 25 mg/L natamycin (#X132, Lab M, UK) at 41.5^°^C, in microaerophilic conditions (5% CO_2_ and 10% O_2_) for 48 h.

**TABLE 1:** Bacteria strains used in this project.

Bacterial strain	Serotype	Source
*Salmonella* Choleraesuis	1, 6,7:c:1,5	DMST 5580
*Salmonella* Dublin	1, 9,12:g,p	DMST 30404
*Salmonella* Enteritidis	1, 9,12:g,m	ATCC 13076
*Salmonella* Hadar	1, 8,z10:e,n,x	DMST 10634
*Salmonella* Infantis	1, 6,7:r:1,5	DMST 26426
*Salmonella* Mbandaka	1, 6,7:z10:e,n,z15	DMST 17377
*Salmonella* Senftenberg	1, 1,3,19:g,s,t	DMST 17013
*Salmonella* Typhimurium	1, 4,12:i:1,2	ATCC 13311
*Salmonella* Virchow	1, 6,7:r:1,2	DMST 32758
*Listeria innocua*	–	DMST 9011
*Listeria ivanovii*	–	ATCC 700402
*Listeria monocytogenes*	–	ATCC 19115
*Listeria welshimeri*	–	DMST 20559
*Escherichia coli*	–	ATCC 25922
*Escherichia coli* O157:H7	–	DMST 12743
*Campylobacter coli*	–	DMST 11353
*Campylobacter jejuni*	–	ATCC 33291
*Staphylococcus aureus*	–	ATCC 25923
*Vibrio parahaemolyticus*	–	ATCC 17802

ATCC, American Type Culture Collection (Manassas, VA); DMST, Department of Medical Sciences, Thailand.

The sources and reactivity of all antibodies and phage clones used in this study were reported in Supplementary Table 1.

### Biopanning and individual phage clone amplification

**FIGURE 1:**
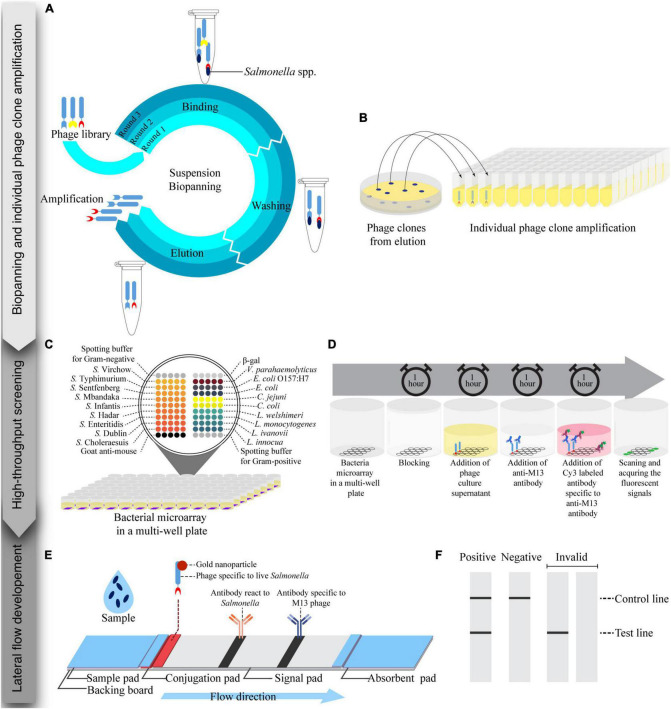
Schematic of the process for the development of a lateral flow strip test assay for live *Salmonella* detection consisting of three steps: biopanning and individual phage clones amplification, high-throughput screening, and lateral flow development. **(A)** Suspension biopanning was performed against a mixture of nine *Salmonella* serovars adapted from Lee et al. (2007). **(B)** Phage clones were amplified in a 96-well plate and screened by a bacterial microarray method. **(C)** A bacterial microarray in a 96-well plate format was developed and constructed. Each well was spotted with target and non-target bacteria. **(D)** Phage supernatant was tested with a bacterial microarray. **(E)** Lateral flow strip test was developed using a gold nanoparticle-labeled phage as a biorecognition element and signal reporter. Anti-*Salmonella* and anti-M13 phage antibodies were printed at the test line and control line, respectively. **(F)** Readouts of lateral flow strip test were visualized by the naked eye and images of the strip were captured by a smartphone.

A phage-displayed human domain antibody library displaying a single human VH framework (V3-23/D47) with diversity introduced in the antigen-binding site and short complementarity-determining region 3 (CDR3) of the heavy chains was used in this study (Lee et al., 2007) (Source Bioscience). Biopanning steps were performed according to the library instructions and modified with a suspension method previously employed (Paoli et al., 2004; Morton et al., 2013a; Figure 1A). Briefly, sterilized protein low binding tubes were blocked with 1 mL of 5% skimmed milk in phosphate buffered saline (PBS, pH 7.4 containing 1 mM KH_2_PO_4_, 0.15 mM Na_2_HPO_4_, 3 mM NaCl) overnight at 4^°^C. The blocked tubes were washed twice with PBS. For the first round of biopanning, a cocktail of nine serovars (Choleraesuis, Dublin, Enteritidis, Hadar, Infantis, Mbandaka, Senftenberg, Typhimurium, and Virchow) of *Salmonella* (5 × 10^9^ colony forming units (CFU)/mL for each *Salmonella* serovar) and the phage library (5 × 10^11^ plaque forming unit, pfu/mL) were mixed in PBS (total volume 1 mL) in the blocked tube, 20 rpm at RT for 1 h. Unbound phages were removed by centrifuging at 3,200 × g for 10 min. The pellet of phage-bound bacterial cells was washed five times by resuspending in PBS containing 0.1% Tween 20 and separation of phage-bound bacterial cell pellet by centrifugation at 3 200 × g for 10 min. To elute phages from the bacterial target, a trypsin solution (1 mL of 100 μg/mL Trypsin in Tris-buffered saline calcium chloride) was added, and the suspension was incubated at RT for 1 h. The eluted phages were used to infect a mid-log phase culture of *E. coli* TG1 TR strain (OD_600_ = 0.5) at 37^°^C for 1 h. The non-infecting phages were separated by centrifugation at 3,200 × g for 5 min. To enumerate the phage-infected *E. coli* TG1, the pellet was resuspended in 1 mL of 2xYT medium, and the bacterial cell suspension was serially diluted and plated on TYE ampicillin glucose agar plates (10 g/L bacto-tryptone, 5 g/L yeast extract, 8 g/L NaCl, 100 mg/L ampicillin and 40 g/L glucose).

To amplify and purify phages for the next rounds of biopanning, the phagemid-carrying *E. coli* TG1 bacterial cells (phage clones) from the TYE agar plates were scraped from plates using 5 mL of 2xYT medium per plate. The bacterial suspension was diluted to an OD_600_ value of 0.1 in 500 mL of 2xYT medium supplemented with 100 μg/mL ampicillin and 4% glucose. Bacteria were cultured at 37^°^C and 250 rpm to an OD_600_ value of 0.5. The bacterial culture was then infected with KM13 helper phages (2 × 10^12^ pfu) and incubated at 37^°^C for 1 h. The supernatant was removed by centrifuging at 3,200 × g for 10 min, and the cell pellets were resuspended in 500 mL of 2xYT medium supplemented with 0.1% glucose, 100 μg/mL ampicillin and 50 μg/mL kanamycin, and incubated at 25^°^C, 250 rpm for 16-20 h. Phages in the supernatant were precipitated with 20% polyethylene glycol 6,000 (20% PEG6000, 2.5 M NaCl) and reconstituted in 1 mL PBS. The phage titer was estimated using the following equation (Lee et al., 2007).


phagetiter(phage/mL)=OD×260dilutionfactor ×22.14×1010

From a phage clone collection, 188 individual colonies of phage-infected cells from each biopanning round were picked from 10-fold serial dilution plates of 2xYT agar supplemented with 100 μg/mL ampicillin and 4% glucose (Figure 1B). Each colony was transferred into a well of 96-well plates (Costar) containing 200 μL of 2xYT medium supplemented with 100 μg/mL ampicillin and 4% glucose, covered with a breathable sealing film (Axygen) and incubated at 37^°^C, 250 rpm overnight. These phage clones were kept at 4^°^C until use.

To screen for specific phage and to test cross-reactivity against other relevant bacteria, 600 μL of 2xYT supplemented with 100 μg/mL ampicillin and 4% glucose was inoculated with 30 μL of a suspension of individual phage clone stock and cultured at 37^°^C, 250 rpm for 3 h. After 3 h of incubation, KM13 helper phages (150 μL of 8 × 10^9^ pfu/mL) were added to infect the cells in the culture, which was then incubated at 37^°^C without shaking for 1 h. To remove the helper phage, the bacterial cells were collected by centrifuging at 2,000 rpm for 30 min. The cell pellets were resuspended in 600 μL of 2xYT supplemented with 100 μg/mL ampicillin, 50 μg/mL kanamycin, and 0.1% glucose and cultured at 25^°^C, 250 rpm for 16-24 h. The phages were separated from bacterial cells by centrifugation at 2,000 rpm for 30 min. The phage supernatants were screened by the optimized bacterial microarray method described below.

### Bacterial microarray development in a multi-well plate

To develop a bacterial microarray method in a multi-well plate (Figures 1C,D), bacterial cells (1 × 10^11^ CFU/mL) were suspended in carbonate buffer (44 mM NaHCO_3_ and 6 mM Na_2_CO_3_, pH 9.6) with Tween 20 (0.05%) and glycerol (0.5%) for Gram-negative bacteria, and in a carbonate buffer with glycerol (0.5%) for Gram-positive bacteria. The bacteria suspension was spotted (5 replicates) onto microplate wells (Corning) using a NanoPrint 210 microarrayer equipped with 946NS pins (TeleChem). Relevant spotting buffers were used as negative controls, and an anti-mouse antibody was used as a positive control and to indicate spot positions. The bacterial microarrays were kept at 4^°^C until use.

### Screening of phage-displayed antibody fragments using a bacterial microarray technique

To identify phage clones specific to *Salmonella* spp., bacterial microarrays were used to screen the phage clones (Figure 1D). The bacterial microarray plates were blocked with 5% skimmed milk in PBST (300 μL/well) for 1 h at RT, then washed with PBST three times by an automatic microplate washer (BIO-RAD). Phage supernatant (100 μL) was added to each well and incubated for 1 h at RT before being washed again as before. An antibody specific to bacteriophage M13 (100 μL/well, 5 μg/mL) was added and the plates were incubated for 1 h at RT. After washing again, a Cy3 labeled anti-mouse antibody (100 μL/well, 5 μg/mL) was added and incubated in the dark for 1 h at RT. The microplates were washed as before and dried by centrifuging at 200 rpm for 5 min. The plates were then scanned using a fluorescence scanner (TECAN), and the fluorescent intensities of spots were determined using the Array-Pro Analyzer software version 4.5.1.73 (TECAN). Background noise in each experiment was determined as the mean value taken from wells containing spotting buffer with no phage added. Normalized signals were determined as the ratio of mean fluorescent intensity of bacterial spots (5 technical replicates/bacterial strain) to background. Mean normalized signals from 5 spots greater than or equal to two were considered positive. To visualize microarray data, a heat map was created from the mean normalized signals with the GraphPad Prism software version 9.4.1 (681).

### Plate-trapped antigen-ELISA

A plate-trapped antigen (PTA)-ELISA method was used to validate the microarray results of the selected phage clones. Bacterial cells (10^9^CFU/mL) were heat inactivated at 100^°^C for 15 min. Each of the 18 inactivated bacterial strains was diluted in carbonate buffer pH 9.6 (1 × 10^8^ CFU/mL, 100 μL/well) and coated onto plate wells overnight at 4^°^C. The plates were then washed by an automatic washer machine (BIO-RAD) with 300 μL/well PBS containing 0.1% Tween 20 (PBST) three times before being blocked with 5% skimmed milk (300 μL/well, Difco laboratory) in PBST for 1 h at RT. The washing step was repeated before 100 μL of phage suspension was added into each well. Plates with phage added were incubated for 1 h at RT. After washing as before, a horseradish peroxidase (HRP)-labeled anti-M13 antibody (diluted 5,000-fold in 5% skimmed in PBST; GE Healthcare) was added and the plate incubated for 1 h at RT. The plate was washed as before and a substrate solution for HRP (TMB: 3,3’,5,5’-Tetramethylbenzidine; Invitrogen) was added (100 μL/well). The plate was then incubated for 5–30 min at RT. The reaction was stopped by adding 0.5 M H_2_SO_4_ (50 μL/well) and the signal was measured at 450 nm absorbance using a SpectraMax M5 microplate reader (Molecular device). Each experiment was repeated three times. A signal three times above the value of background reading was considered positive.

### Limit of detection of phage clones by plate-trapped antigen-ELISA

Twelve different bacteria titers (0, 1 × 10^4^, 5 × 10^4^, 1 × 10^5^, 5 × 10^5^, 1 × 10^6^, 5 × 10^6^, 1 × 10^7^, 5 × 10^7^, 1 × 10^8^, 5 × 10^8^, and 1 × 10^9^ CFU/mL) were prepared by dilution of bacterial culture in carbonate buffer. The bacteria were coated onto plate wells. The assay was performed in triplicate using the same steps as described above in the PTA-ELISA method. Limit of detection (LOD) values were calculated as the minimal titer with a signal greater than three times background (negative control). The ELISA signal data were fitted to the following dose-response equation with three parameters and confidence level at 95% (Iturria, 2005; Charlermroj et al., 2014).


%B/B=0[B+0(B-B)0/(1+10)LogEC50-x]×100,

where B and B_0_ were absorbance values at 450 nm of a phage clone binding in the presence (B) or absence (B_0_) of bacterial cells, respectively, in which% B/B_0_ response was measured at varying titers of bacteria (X), and EC_50_ is the bacterial titer that produced a 50% response between B and B_0_.

### Preparation of whole phage-gold nanoparticles (phage-AuNPs) conjugates

To prepare phage-AuNPs, AuNPs solution (1 mL, 40 nm, #KP-05120003, Kestrel Bioscience, Thailand) was adjusted to pH 8.0 with 100 mM K_2_CO_3_ before purified whole phage (100 μL of 10^12^ pfu/mL) suspension was added. The mixture was incubated at RT for 10 min. The unconjugated AuNPs were subsequently blocked with bovine serum albumin (BSA, 110 μL of 10% (w/v) in distilled water adjusted to pH 7.0) at 4^°^C overnight. The excess of phage was removed by centrifugation at 12,000 rpm at 4^°^C for 30 min. The pellet was resuspended in 50 μL of conjugate buffer (PBS containing 10% sucrose, and 5% trehalose, pH 7.4). The phage-AuNPs suspension was kept at 4^°^C until use.

### Preparation of lateral flow test strips

The composition of lateral flow test strips is shown in Figure 1E. A sample pad (CF3, GE Healthcare, USA) was impregnated with PBS containing 0.4% Tween 20, and 2% (w/v) BSA, pH 7.4, before drying overnight at 37^°^C. The suspension of whole phage-AuNPs conjugates (10 μL) was applied on a conjugated pad (4 mm × 1 cm., GF33, Kestrel Bioscience, Thailand), and dried at 37^°^C for 30 min. An antibody specific to *Salmonella* (1 mg/mL, ab35156, AbCAM, UK) and an antibody specific to M13 phage (0.5 mg/mL) were prepared in carbonate buffer, pH 9.6. The antibodies were dispensed (0.8 μL/cm) on the signal pad (CN95, Kestrel Bioscience, Thailand) at a test line (TL) for *Salmonella* detection and a control line (CL) for positive control, respectively, using a non-contact microarray dispenser equipped with Biojet Elite dispenser (AD1520, BioDot, USA). The signal pad was then dried at RT for 30 min before blocking with treating buffer (10 mM di-sodium tetraborate containing 1% (w/v) BSA, 0.5% (w/v) polyvinyl pyrrolidone (PVP40), and 0.15% (v/v) Triton X-100, pH 8.0) and dried at 37^°^C overnight. The sample pad, conjugate pad, and absorbent pad (CF5, GE Healthcare, USA) were assembled onto a backing board, and cut into 4 mm wide strips with a guillotine cutter (CM5000, BioDot, USA).

### Lateral flow assay procedure

The lateral flow test strips were tested as follows. Bacteria were cultured in Luria-Bertani broth (LB, Difco, USA) at 37^°^C, 250 rpm for 16-18 h. Test sample (100 μL) was applied onto the sample pad, and incubated for 15 min at RT. Signals were captured at 11 cm above the strip by a smart phone (Samsung Note 20) under the white light condition for qualitative assessment of the result (Figure 1F). To test the specificity of the lateral flow test strips, nine serovars of *Salmonella*, heat-killed *Salmonella* Enteritidis, and three other bacteria strains (*E. coli* O157:H7, *L. monocytogenes*, and *S. aureus*) were tested at 10^8^ CFU/mL with three replications. For sensitivity of detection, ten different titers of *Salmonella* Enteritidis (0, 1 × 10^6^, 5 × 10^6^, 1 × 10^7^, 5 × 10^7^, 1 × 10^8^, 5 × 10^8^, 1 × 10^9^, 5 × 10^9^, 1 × 10^10^ CFU/mL were tested with three replications. To analyze optical density of test line (TL) and control line (CL), the images were converted to grayscale using the Adobe Photoshop Software version 23.5.0, and band intensities were subsequently measured using the Quantity One Software version 4.6.8. The TL/CL ratio values were calculated and used for a statistical analysis.

### Statistical analysis

All data are express as the mean values ± standard deviations (mean ± *SD*) of three replicates, except a specificity test by an ELISA method. All analyses were conducted using the GraphPad Prism 9 (681) software. The experiments were compared using an analysis of variance (ANOVA) and Tukey’s post-test. *P*-value of < 0.05 was considered to indicate a significant difference.

## Results

### Phage high-throughput screening and characterization

To facilitate the screening of phage binders specific to bacteria of interest, a bacterial microarray was developed. Microarray spotting buffers were first optimized using *Salmonella* Typhimurium and *Listeria monocytogenes* as model cells. We found that a carbonate buffer containing Tween 20 and glycerol (CBTG) and a carbonate buffer containing glycerol (CBG) was suitable for Gram-negative (Supplementary Figure 1A) and Gram-positive bacteria, respectively (Supplementary Figure 1B). These spotting buffers were validated with 18 different bacterial strains using antibodies and a phage with known specificities (Supplementary Table 1). The antibodies and phage showed accurate detection of their corresponding bacterial targets when the bacterial cells were prepared in the selected spotting buffers, except in case of SalKPL and ListKPL antibodies (Supplementary Figure 1C). SalKPL antibody reacted strongly with *Salmonella* spp. with some cross reactivity with *E. coli* in the bacterial microarray. This result is in agreement with cross reactivity to related Enterobacteriaceae reported by the antibody’s supplier. ListKPL showed minor cross reactivity to *Salmonella* Infantis on the microarray, which agrees with the cross-reactivity information from the antibody supplier. The results showed that they were suitable for the production of bacterial microarray.

**FIGURE 2:**
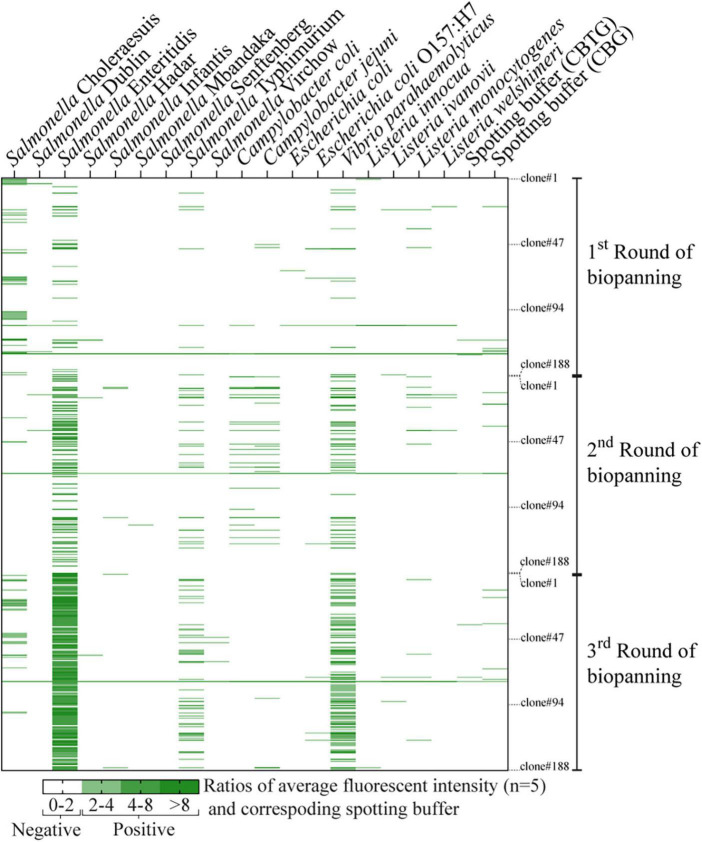
Heat map of individual phage clones (rows) tested against 18 different bacteria strains (columns). A total of 564 phage clones (188 clones for each round of biopanning, 3 rounds total) were tested for their binding specificity using bacterial microarray. Fluorescent intensity values were averaged from five spots and normalized with the signal from their corresponding spotting buffers (CBTG for Gram-negative bacteria, and CBG for Gram-positive bacteria). Normalized fluorescent signals greater than or equal to two were considered as positive results and are indicated by the green color gradient. Normalized signals (negative results) below the threshold are indicated in white.

**FIGURE 3:**
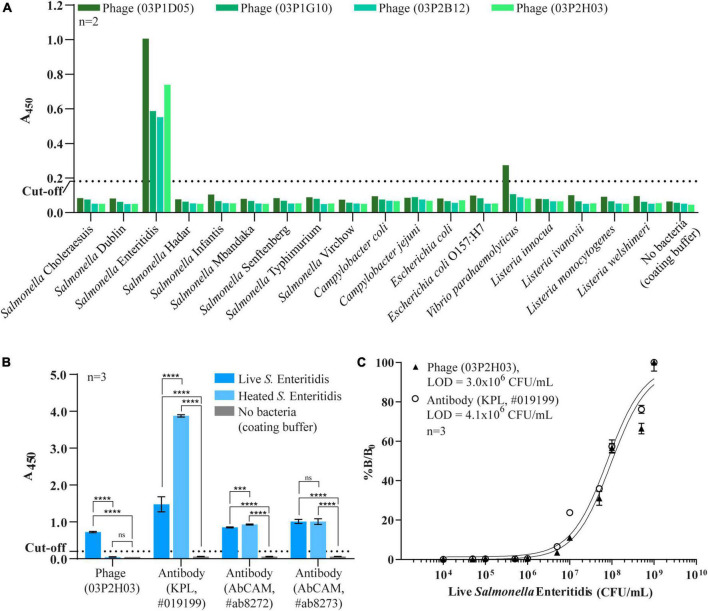
Characterization of randomly selected phage clones by a PTA-ELISA method against 18 different strains of bacteria. **(A)** Specificity of the four phage clones previously selected from bacterial microarray data. **(B)** Comparison of specificity against live/dead cells of the selected phage clone 03P2H03 and the three commercial antibodies specific to *Salmonella*. Each bar is represented and mean ± standard derivation (*SD*) which was compared to no bacteria (one-way ANOVA for indicate comparison, Tukey’s multiple comparison test, ***p < 0.005, ****p < 0.0001, and *ns* = no significance). **(C)** Limit of detection (LOD) for live *Salmonella* Enteritidis of phage clone (03P2H03) and a commercial *Salmonella* antibody by PTA-ELISA. The dotted line represents the cut-off value three times that of the background (LB medium) used to determine the limit of detection. Error bars indicate standard derivation (SD) (*n* = 3). Solid lines indicate curve fits to the data using a three-parameter dose-response model.

The bacterial microarray was used to screen a total of 564 phage clones (188 from each round of biopanning, 3 rounds in total) and to test the specificity of the phage-displayed antibody against 18 different bacterial strains. The number of *Salmonella-*specific phage clones increased after three rounds of biopanning (20.2, 20.2, and 35.6% for the 1st–3rd biopanning rounds, respectively), while the number of phage clones that could bind to *Salmonella* and cross-react with other bacteria dramatically increased (8.5, 22.3, and 57.4% for the 1st–3rd biopanning rounds, respectively) (Figure 2). We sought phage clones that can detect the nine serovars of *Salmonella* with no cross-reactivity to other bacterial species. Unfortunately, no phage clones were isolated that bound to all nine serovars of *Salmonella* after the third round of biopanning. Moreover, most of the phage clones detecting one or more serovars of *Salmonella* also cross-reacted with *Vibrio parahaemolyticus*. However, we were able to identify phage clones specific to *Salmonella* Enteritidis. Thus, we randomly selected four phage clones from the third round of biopanning for further characterization using a PTA-ELISA method against 18 different bacterial strains (Figure 3A). All randomly selected phage clones could specifically bind to *Salmonella* Enteritidis, except for clone 03P1D05 that showed some cross-reactivity to *V. parahaemolyticus* (Figure 3A). Of the remaining three *Salmonella*-specific phage clones, clone 03P2H03 exhibited the highest signal (Figure 3A) and was selected for its ability to discriminate between live and dead *Salmonella*. 03P2H03 was able to distinguish between live and dead (heat-killed) *Salmonella* Enteritidis cells, while the commercial antibodies could not (Figure 3B). The limit of detection (LOD) of the 03P2H03 phage clone in detecting *Salmonella* was found to be within the same order of magnitude (3.0 × 10^6^ CFU/mL) as that of the *Salmonella*-specific antibody (4.1 × 10^6^ CFU/mL) (Figure 3C).

### Lateral flow test strip for detection of live *Salmonella* Enteritidis

**FIGURE 4:**
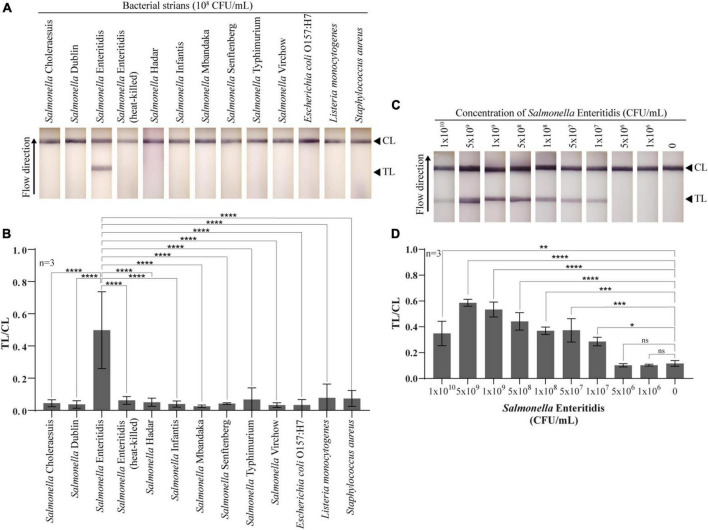
Specificity and sensitivity of the developed lateral flow test strip for *Salmonella* Enteritidis detection. **(A)** Photographs of the lateral flow test strips with different 12 bacterial strains and heat-killed *Salmonella* Enteritidis at 10^8^ CFU/mL in LB medium. **(B)** Quantitative of lateral flow test strips for specificity test. Test line (TL) and control line (CL) ratios were compared with *Salmonella* Enteritidis using a statistical analysis. **(C)** Photographs of the lateral flow test with ten different titers of *Salmonella* Enteritidis. **(D)** Quantitative of lateral flow test strips for sensitivity test. Test line (TL) and control line (CL) ratios were compared with no bacteria using a statistical analysis. All experiments were tested with three independent test strips (one-way ANOVA for indicate comparison, Tukey’s multiple comparison test, *p < 0.05, **p < 0.001, ***p < 0.005, ****p < 0.0001, and *ns* = no significance).

To test the specificity and sensitivity of the constructed lateral flow test strip using whole phage-AuNPs as a detecting molecule, different bacterial strains and varying titers of live *Salmonella* Enteritidis were tested. The lateral flow test strip could specifically detect *Salmonella* Enteritidis but showed no reaction to eight *Salmonella* serovars and other relevant bacterial strains (Figures 4A,B). For sensitivity, the test line signal indicating the presence of *Salmonella* Enteritidis was apparent to the naked eye for cell titers 1 × 10^7^–1 × 10^10^CFU/mL, indicating a LOD of 1 × 10^7^ CFU/mL for the test strip. At the highest titer tested (1 × 10^10^CFU/mL), the test line showed slightly lower intensity than lower titers (Figures 4C,D). The assay time of the lateral flow test strip was only 15 min. These results demonstrated the usefulness of whole phage as a biorecognition element for a lateral flow detection method of live *Salmonella* Enteritidis.

## Discussion

While phage technology can be applied for pathogen detection, its full potential has yet to be realized owing to the difficulty in obtaining specific phage clones for downstream applications. In this study, a high-throughput microarray method was developed to screen and characterize the binding specificity of phage clones. From prior experience, we found that selection of appropriate spotting buffers was the key factor for success. For instance, we found that a carbonate-based buffer was suitable for producing an antibody array (Charlermroj et al., 2014). Unlike the antibody array, the bacterial microarray in this study is more complicated because the major components of the bacterial cell wall for each Gram stain reactive group are different, i.e., lipopolysaccharide for Gram-negative bacteria and peptidoglycan for Gram-positive bacteria. Thus, spotting buffers needed to be optimized for each type of bacterial cell.

The bacterial microarray in multi-well plate format provides several advantages. First and foremost, the microarray was able to identify phages specific to *Salmonella* from the first round of biopanning. Generally, most protocols recommend 3–5 rounds of biopanning to obtain specific phage clones (Song et al., 2011; Hamzeh-Mivehroud et al., 2013). However, when a phage-antibody library is selected against highly complex targets such as whole cells, a strong bias for binders against abundant proteins has been reported. Therefore, the ability to characterize binders early in the biopanning process will result in a wider diversity of binders that can be used for diagnostics development (de Wildt et al., 2000).

The selected phage clone reacts to *Salmonella* Enteritidis, but did not react to eight serovars of *Salmonella*. While *Salmonella* Enteritidis and *Salmonella* Dublin belong to the same group of O antigen (D1 group), these two serovars have different H antigens: g and m for *Salmonella* Enteritidis, and g and p for *Salmonella* Dublin (Grimont and Weill, 2007). It might be that this phage clone bind to flagella “m” antigen of *S*. Enteritidis, resulting in specificity to only *Salmonella* Enteritidis among the nine *Salmonella* serovars, which should be investigated further in the next research.

The phage-derived antibody fragments identified by the microarray method showed similar characteristics (LODs and specificity) to those of the commercial antibodies. Moreover, the selected phage clone was able to distinguish between live and dead cells, whereas commercial antibodies cannot. Many publications reported the use of phages as biorecognition elements for the diagnosis of *Salmonella* (Morton et al., 2013b; Karoonuthaisiri et al., 2014); however, to our knowledge, there is no report on phages that can discriminate live from dead *Salmonella* Enteritidis.

The fact that our phage-derived antibody fragments were able to discriminate between viable and dead cells can be instrumental for many applications, especially for food safety. Molecular techniques have been developed to discriminate between viable and dead cells, including PCR-based methods such as reverse transcription (RT)-PCR and nucleic acid sequence-based amplification (NASBA) (Morin et al., 2004), and viability dyes coupled with DNA amplification (Elizaquivel et al., 2013). These molecular techniques are often more tedious and laborious than immunoassay-based methods and are typically only employed in a laboratory. Therefore, the phage-derived antibody fragments that can distinguish viable from dead foodborne pathogens can open a new horizon for more rapid and simpler diagnostics for food safety, which can be applied in different settings outside of the laboratory.

We developed a lateral flow test strip using the selected phage as a biorecognition element for the detection of live *Salmonella* Enteritidis and proved the specificity of the assay for detecting *Salmonella* Enteritidis. In addition, although this phage clone was not previously screened against *Staphylococcus aureus* during the screening process, the test strips using this phage showed no cross-reactivity with *S. aureus*. The low intensity observed at the highest titer of *Salmonella* (1 × 10^10^ CFU/mL) could be explained by the hook effect from excessively high antigen concentration in lateral flow immunoassays (Ross et al., 2020). Although sensitivities of the phage clone and antibody were around 10^6^ CFU/mL by the PTA-ELISA method, the sensitivity for the phage-based lateral flow test strip was found to be 10^7^ CFU/mL. The greater sensitivity of the ELISA method compared with that of the lateral flow method has previously been reported (Serrano et al., 2020). However, the sensitivity of the phage-based lateral flow test strip in this study is lower than that of a previous report of an immuno-lateral flow assay for *Salmonella* Enteritidis and Typhimurium at 10^6^ and 10^4^CFU/mL in culture media, respectively (Moongkarndi et al., 2011). The sensitivity of the test strip can be further improved by exploring other reporting molecules such as luminescent nanoparticles (Xie et al., 2014), carbon nanoparticles (Noguera et al., 2011), and streptavidin-labeled AuNPs (Chen et al., 2022).

In summary, a lateral flow test strip assay developed using phage from microarray screening was able to distinguish live from dead *Salmonella* Enteritidis. The assay time of 15 min is much shorter than that of any culture-based method which usually requires at least 24 h. The microarray-based screening method presented here is not limited to the selection of *Salmonella*-specific phage-derived binders, but it can also be readily adapted for phage-based binder selection against other to other pathogen targets, obviating the need for animal immunization. In addition, phage-based lateral flow test strip assay is not limited to foodborne pathogens and could be applied to other targets of interest, e.g., viruses.

## Data availability statement

The original contributions presented in this study are included in the article/Supplementary material, further inquiries can be directed to the corresponding author/s.

## Author contributions

RC and NK conceived the idea, designed the experiments, and wrote the manuscript. RC, MM, and SP carried out the experiments. All authors contributed to the data analysis and reviewed the manuscript.
