# Supplementary material for: A rapid colorimetric lateral flow test strip for detection of live Salmonella Enteritidis using whole phage as a specific binder
Source: Front Microbiol. 2022 Sep 29;13:1008817. doi: 10.3389/fmicb.2022.1008817 (PMC9556839; doi:10.3389/fmicb.2022.1008817)
Supplement: Supplementary file 1 [file Data_Sheet_1.DOCX]

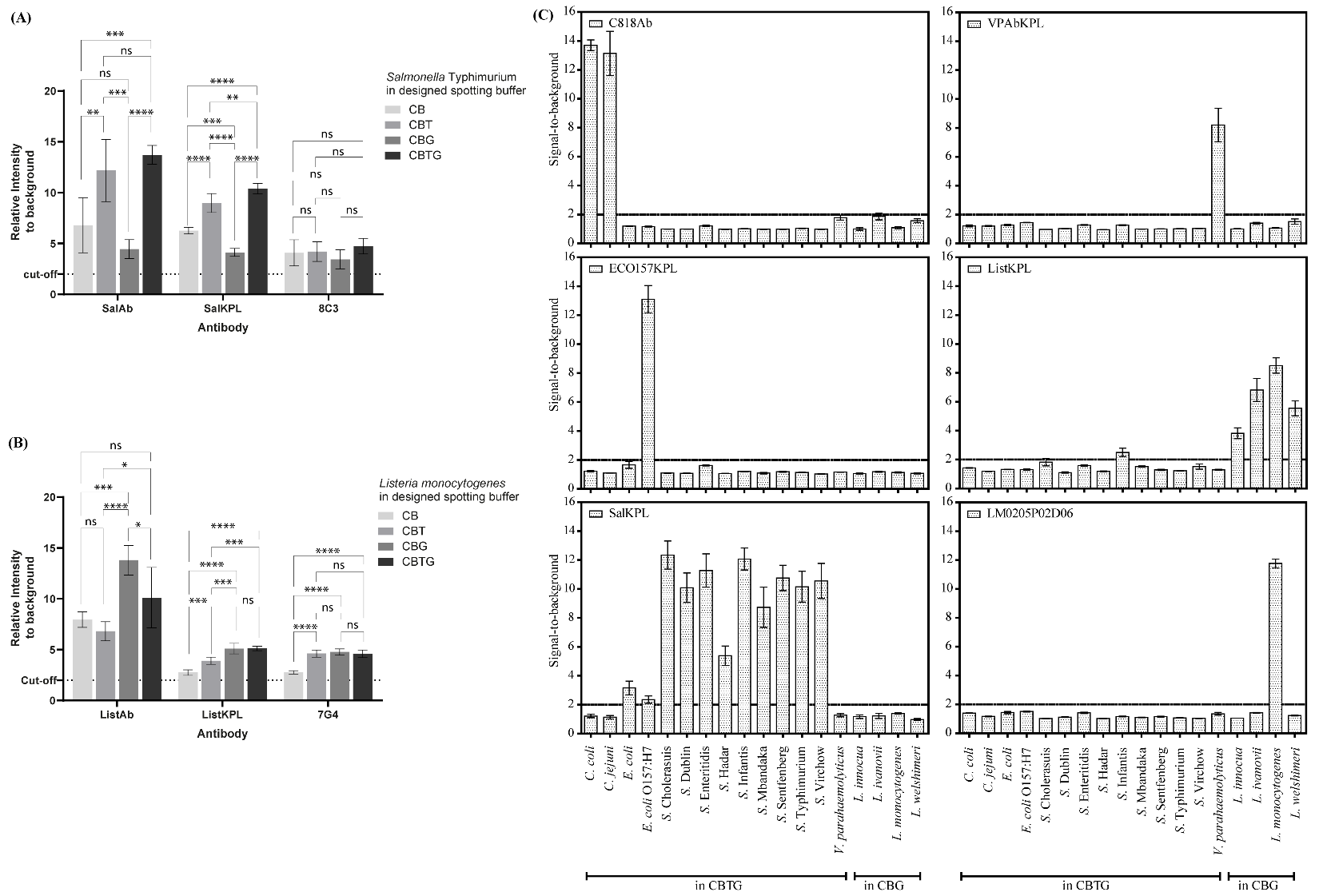


**Supplementary Figure 1**. Spotting buffer selection for bacterial microarray development. Carbonate buffers (CB) containing eight different combinations of Tween 20 (T) and/or glycerol (G) were tested with (A) *Salmonella* Typhimurium, a representative of Gram-negative bacteria, and (B) *Listeria monocytogenes*, a representative of Gram-positive bacteria. (C) A bacterial microarray developed using CBTG and CBG spotting buffers for the Gram-negative and Gram-positive bacteria, respectively, tested with antibodies specific to *Campylobacter* spp.(C818Ab), *E. coli* O157:H7 (ECO157Ab), *Salmonella* spp. (SalKPL), *Vibrio parahaemolyticus* (VPAb), *Listeria* spp. (ListKPL), and whole phage specific to *Listeria monocytogenes* (LM0205P02D06 phage). Dotted lines represent cut-off values which indicate a value twice that of the background. Error bars indicate standard derivation (SD) values (n=5). **p* < 0.05, ***p* <0.005, ****p* < 0.0005, *****p* < 0.0001, and *ns* = non-significantly different.
