# Supplementary material for: A rapid colorimetric lateral flow test strip for detection of live Salmonella Enteritidis using whole phage as a specific binder
Source: Front Microbiol. 2022 Sep 29;13:1008817. doi: 10.3389/fmicb.2022.1008817 (PMC9556839; doi:10.3389/fmicb.2022.1008817)
Supplement: Supplementary file 2 [file Data_Sheet_2.DOCX]

**Supplementary Table 1.** Antibodies and peptide-displayed phage used in this study

| **Antibody/Phage** | **Type** | **Reactivity** | **Source/Reference** |
| --- | --- | --- | --- |
| C818Ab | MAb | *Campylobacter* spp. | USDA |
| VPAb | PAb | *Vibrio* spp. | KPL (#019002) |
| ECO157Ab | PAb | *E. coli* O157:H7 | KPL (#019590) |
| SalKPL | PAb | *Salmonella* spp. | KPL (#019199) |
| SalAb | MAb | *Salmonella* spp. | Abcam (#8273) |
| 8C3 | MAb | *Salmonella* spp. | BIOTEC |
| ListKPL | PAb | *Listeria* spp. | KPL (#019090) |
| ListAb | MAb | *Listeria monocytogenes* | Abcam (#11438) |
| 7G4 | MAb | *Listeria monocytogenes* | BIOTEC (Charlermroj, et al., 2012) |
| LM0205P02D06 | Peptide-displayed phage | *Listeria monocytogenes* | BIOTEC and QUB (Morton, et al., 2013) |
| Cy3 labeled anti-mouse antibody | PAb | Mouse antibodies | KPL (#072-01-18-09) |
| Cy3 labeled anti-rabbit antibody | PAb | Rabbit antibodies | Zymed (#81-6115) |
| Anti-M13 mouse antibody | MAb | M13 bacteriophage | GE-healthcare (27-9420-01) |
| Anti-mouse antibody | PAb | Mouse antibodies | KPL (#5210-01-87) |

*MAb: Monoclonal antibody*

*PAb: Polyclonal antibody*

*USDA: U.S. Department of Agriculture*

*KPL: Kirkegaard and Perry Laboratory Inc., USA*

*Abcam: Abcam Inc., UK*

*BIOTEC: National Center for Genetic Engineering and Biotechnology, Thailand*

*QUB: Queen’s University Belfast, UK*
